# Preventing varicella zoster infection in immunocompromised adults with varicella zoster–specific immunoglobulins

**DOI:** 10.1017/ash.2023.167

**Published:** 2023-05-26

**Authors:** Daniel Gelman, Miri Zektser, Lior Nesher

**Affiliations:** 1 Infectious Disease Institute, Soroka University Medical Center, Ben-Gurion University of the Negev, Beer Sheva, Israel; 2 Department of Military Medicine, Faculty of Medicine, The Hebrew University of Jerusalem, Jerusalem, Israel; 3 Department of Hematology, Soroka University Medical Center, Beer Sheva, Israel

## Abstract

**Background:**

Varicella zoster virus (VZV) exposure seriously threatens immunocompromised hosts. Postexposure prophylaxis (PEP) using immune globulins is considered the standard of care; however, the available literature is mainly based on its use in pediatric patients. Here, we describe a widespread VZV exposure among immunocompromised adults treated with VZV-specific immunoglobulins (VZVSIG), and we discuss management and outcomes.

**Methods:**

We conducted a retrospective study to describe the exposure of immunocompromised patients to a single healthcare worker with primary VZV in 2019. Patients were grouped by their overall risk for infection, and those at risk received a single intramuscular dose of 625 IU of VZVSIG and were followed for 1 year.

**Results:**

In total, 83 patients received PEP at <96 hours of exposure: 14 were hospitalized, 68 were outpatients, and 1 was an immunocompromised staff member. The median age was 69 years (range, 21–92), and 49.4% were male. In addition, 30% of the patients were deemed high risk, 42% were intermediate risk, and 28% were considered low risk, although they were given PEP. Varicella infection was not diagnosed in any patient in the first weeks of follow-up. However, during the year of follow-up, 4 patients developed symptoms suspicious of VZV, all >3 months after exposure, thus were probably unrelated to the event. Adverse events related to VZVSIG (pyrexia) were reported in 2 patients (2.4%).

**Conclusions:**

Our findings demonstrate the utility of VZVSIG as PEP in one of the largest cohorts of immunocompromised adults to date. No early varicella infection was found following exposure, supporting the current recommendations of the VZVSIG administration.

Varicella zoster virus (VZV) infection poses a serious threat to immunocompromised hosts,^1,2^ with increasing reports of fatal complications in this population.^3–5^ Several postexposure prophylaxes (PEP) regimens for VZV are currently available.^6^ Among them, passive immunization is considered the standard for immunocompromised hosts with hematologic malignancies or systemic immunosuppressive therapies.^6,7^

Several varicella zoster human-specific immunoglobulin (VZVSIG) products are currently available worldwide. According to the US Centers for Disease Control and Prevention (CDC), VZVSIG is recommended for at-risk patients to be administered as early as possible.^7,8^ However, due to recent revisions in the available VZVSIG products and the focus of current publications on the pediatric population,^9,10^ additional evidence regarding the role of the newer products in immunocompromised adults is needed. Here, we describe a large-scale VZV exposure incident that was controlled by the rapid administration of the newer VZVSIG.

## Methods

We conducted a retrospective study to describe the exposure of 83 immunocompromised patients in the hematological unit of Soroka University Medical Center to a healthcare provider (HCP) with primary varicella infection. The exposure event occurred in 2019 when a physician in advanced training developed a fever and rash diagnosed as acute VZV while on call overnight at the hematological-transplant unit after a full day of examining patients in the clinic and transfusion center. Following the diagnosis of acute VZV, an epidemiological nurse used electronic medical records to identify all patients with whom the HCP came into contact. Meaningful exposure was considered indoor face-to-face contact with the infected HCP for >5 minutes, starting 24 hours before symptoms appeared.^11^ Following a careful evaluation by a hematologist, patients identified as immunocompromised based on clinical criteria were provided PEP in the form of a single IV dose of 12.5 units/kg (maximum dose, 625 units) of VZVSIG (Varitect CP, GmbH, Germany).

Following approval and waiver of informed consent from the institutional ethics board in research (SOR-20-059), we accessed all identified patients’ electronic medical records. We collected data on the demographics and medical histories of these patients, as well as data on their exposures, the treatments provided, and the long-term outcomes up to a year following the event. The treated patients were grouped by their overall VZV infection risk on the date of exposure according to the level of immunosuppression, as determined by the NCCN guidelines for the prevention and treatment of cancer-related infections.^12^ In specific cases not addressed by the NCCN guidelines, risk stratification was performed according to clinical assessment by a hematologist and an infectious disease expert. Patient outcomes, including clinical manifestations of VZV infection and all-cause mortality, were analyzed in all groups.

## Results

In total, 83 patients received PEP. The baseline characteristics of these patients at the time of exposure and their stratification according to the risk of infection based on their level of immunosuppression are shown in Table 1. Moreover, 14 patients were hospitalized at the time of exposure; 68 were outpatients; and 1 was an immunocompromised staff member. Among all patients, 49.4% were male, and the median age was 69 years (range, 21–92).

**Table 1.** Patient Characteristics and Outcomes Following IV VZV-Specific Immunoglobulins Administration for Postexposure Prophylaxis, Stratified by the Proposed Risk of Infection

Variable		VZV Infection Risk Stratification			
		Total	High	Intermediate	Low
Patients		83	25 (0.301)	35 (0.422)	23 (0.277)
Age	Median	69	65	75	70
	Range	21–92	28–86	21–92	34–87
Sex	Male	41 (0.494)	11 (0.44)	20 (0.571)	10 (0.435)
	Female	42 (0.506)	14 (0.56)	15 (0.429)	13 (0.565)
Location	Inpatient	14 (0.169)	7 (0.28)	7 (0.2)	0
	outpatient	69 (0.831)	18 (0.72)	28 (0.8)	23 (1)
Timing of PEP	<48 h	51 (0.614)	18 (0.72)	27 (0.77)	6 (0.26)
	48–72 h	30 (0.361)	6 (0.24)	8 (0.23)	16 (0.695)
	72–96 h	2 (0.024)	1 (0.04)	0	1 (0.043)
Adverse events		2 (0.024)	2 (0.08)	0	0
Suspected VZV		0	0	0	0
Late report of VZV (>3 m)		4 (0.048)	0	4 (0.114)	0
Survival at 1 y of follow-up		68 (0.903)	16 (0.64)	29 (0.828)	23 (1)
			Risk Factor (No. of Patients)		
			Allogeneic HSCT (9) ^a^ Plasma cell disorders^b^ with immunosuppressive therapy^c^ (8) Acute leukemia (5) Other specified immunosuppressive therapy^d^ (3)	Lymphoma^e^ with immunosuppressive therapy (22) MDS with immunosuppressive therapy (8) Autologous HSCT (4) Past allogeneic HSCT^f^ (1)	MDS/MPN^g^ (9) Lymphoma^e^ without immunosuppressive therapy (6) MGUS (3) Neutropenic disorder (2) Steroid use (2) Solid tumor (1)

Note. VZV, varicella zoster virus; HSCT, hematopoietic stem cell transplant; MDS, myelodysplastic syndromes; MPN, myeloproliferative neoplasm; MGUS, monoclonal gammopathy of undetermined significance; GVHD, graft versus host disease.

a <1 year from transplantation or with evidence of GVHD.

b Multiple myeloma and plasmoblastic myeloma.

c Including proteasome inhibitors or monoclonal antibodies.

d Phosphoinositide 3-kinase delta inhibitors or Janus kinase inhibitors.

e Hodgkin lymphoma, non-Hodgkin lymphoma, or chronic lymphocytic leukemia.

f >1 year from transplantation, without immunosuppression or GVHD.

g Including MDS without immunosuppressive therapy, chronic myeloid leukemia, mastocytosis, and other MPNs.

Overall, 25 patients (30%) were at high risk of infection. These included patients who underwent allogeneic hematopoietic stem cell transplant (HSCT) within the last year and those suffering from graft versus host disease (GVHD); patients treated for plasma cell disorders, including proteasome inhibitors or monoclonal antibodies; patients suffering from acute leukemia; and recipients of recent immunosuppression by PI3K δ or Janus kinase inhibitors. Moreover, 35 patients (42%) were deemed to have intermediate infection risk, consisting mainly of patients treated for lymphomas, myelodysplastic syndrome (MDS), or chronic lymphocytic leukemia. This group also included autologous HSCT recipients and allogeneic HSCT recipients for whom >1 year had passed since transplantation and who had not developed GVHD. Also, 23 patients (28%) were relatively low risk: patients with various hematologic conditions not currently receiving immunosuppressive treatments, including MDS or myeloproliferative neoplasms and lymphomas; patients diagnosed with monoclonal gammopathy of undetermined significance; patients with otherwise unspecified neutropenia; patients receiving long-term treatment with steroids; and patients recently receiving recent solid-organ transplantation.

Except for administering VZVSIG for PEP, no changes were made to the regular medication regimens. Overall, 8 patients received concomitant antiviral prophylaxis, including those with acute leukemia receiving induction chemotherapy and those undergoing an HSCT procedure. These patients continued long-term prophylaxis with oral acyclovir (400 mg twice daily).

PEP was administered within the first 96 hours from exposure. Overall, 51 patients (61%) were treated within 48 hours and 81 patients (97.6%) were treated within 72 hours. All patients were followed for the first few months either while they were admitted or at the hematologic outpatient clinics either physically or by phone. Among 68 patients who survived for 1 year following the exposure, 63 (92.6%) completed at least 1 long-term visit 6 months after the event.

### Outcomes of exposure and PEP

**Fig. 1.**
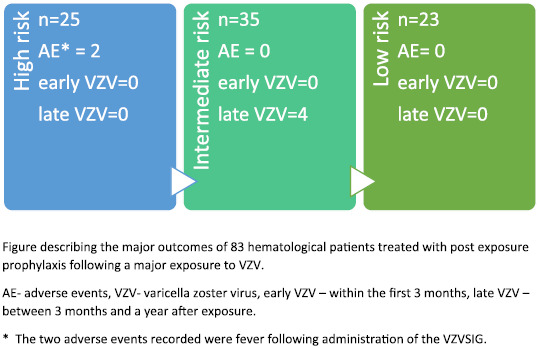
Chart describing the outcomes of patients according to risk.

The outcomes and adverse events following PEP administration are summarized in Table 1. Varicella infection was not suspected in any patient during the initial follow-up month. However, 4 patients developed symptoms suspicious of possible VZV infection during the long-term follow-up, all from the intermediate-risk group (Fig. 1). All of these cases were diagnosed >3 months following exposure. Their charts were reviewed by an infectious disease physician and the events were determined to be reactivation of VZV. Thus, in our opinion, these cases were unrelated to the exposure to HCP.

Adverse events (AEs) that were considered to be related to immunoglobulin administration were reported in 2 patients (2.4%). Both patients belonged to the high infection risk group due to allogeneic HSCT in their first year after transplantation, and both suffered from pyrexia. No other AEs related to PEP have been reported.

## Discussion

This report demonstrates the role of VZVSIG as PEP in one of the largest cohorts of immunocompromised adults published to date. Only a few publications have addressed the utility of such preparations, with minimal data in this population.^9,10^ In one of the most extensive studies published to date describing the use of VZVSIG in a 6-year, open-label, expanded-access program from 285 clinical study sites across the United States, only 263 immunocompromised participants were included in the efficacy assessment, among whom only 32 were adults.^10^ In that study, the varicella rate after VZVSIG prophylaxis was 3.1% among immunocompromised adults, with no cases occurring among patients treated within 96 hours of exposure. A subgroup analysis published in January 2021^9^ reported a 6% incidence of varicella among this population, with 10% for immunocompromised oncologic patients and without any varicella-related complications. The rates of varicella after VZVSOG in the current study were lower than those in these recent reports, even though the administered immunoglobulin dose was lower than previously recommended.^13^

In addition, our study presents a real-world VZV infection risk stratification of hemato-oncologic patients, considering various conditions and treatment regimens. In the previously described subanalysis of immunocompromised patients from the VariZIG expanded-access program,^9^ the patients were stratified based on oncologic immunodeficiencies, primary immunodeficiencies, solid-organ transplant recipients, hematopoietic cell transplant recipients, and other or unspecified conditions. The suggested categorization in this study provides a considerably more comprehensive and practical approach to risk stratification, which could be further used in research and clinical practice.

As a retrospective study, possible incomplete patient medical records reports may have affected our results. Thus, we addressed this limitation by analyzing repeated follow-up visits of patients after the event, at the same medical center, for >1 year. One of the limitations is the lack of controls; consequently, we cannot definitively comment on efficacy and safety of this product. However, our clinical experience in a real-world setting provided a practical evaluation of the role of VZVSIG in the prevention of varicella infection in immunocompromised adults with diverse medical histories. These data provide additional support for the current recommendation of passive immunization as PEP for this population.
